# Numerical study of the structural design influence on cartilage cell differentiation in mechanically stimulated hydrogel scaffolds using an FSI-based model

**DOI:** 10.1007/s10237-025-01976-1

**Published:** 2025-06-15

**Authors:** Pedram Azizi, Christoph Drobek, Hermann Seitz

**Affiliations:** 1 Chair of Microfluidics, Faculty of Mechanical Engineering and Marine Technology, University of Rostock, Rostock, Germany; 2 Chair of Electromagnetic Field Theory, Faculty of Computer Science and Electrical Engineering, Institute of General Electrical Engineering, University of Rostock, Rostock, Germany; 3 Department of Life, Light and Matter, University of Rostock, Rostock, Germany

**Keywords:** Cell differentiation, Tissue engineering, Fluid–structure interaction, Computational fluid dynamic, Finite element analysis, In silico

## Abstract

Three-dimensional (3D) hydrogel scaffolds show considerable promise for the regenerative treatment of cartilage and bone defects. Within tissue engineering, these scaffolds can be mechanically stimulated to specifically promote cartilage formation. While in vitro experiments are traditionally used to study the influence of scaffold structure on cell differentiation, in silico studies offer a complementary, cost-effective, and powerful approach. This numerical study employs a transient fluid–structure interaction (FSI) model to modify the structural design of a mechanically stimulated hydrogel scaffold for enhanced cartilage cell differentiation. The study involved two key modification steps applied to scaffolds under 5% compression. In the first step, scaffold porosity was adjusted by altering the number of strands per layer. The scaffold designed with 38% porosity, consisting of 9 strands per layer across 9 layers, improved cartilage differentiation by approximately 15%. The second step focused on scaling the selected scaffold from step 1 by adjusting the number of layers while keeping the porosity constant, aiming to optimize pore dimensions. This led to a slight improvement in cartilage differentiation of about 2.3%. The results indicate that porosity exerts a more significant influence on cell differentiation than pore size in the structured scaffolds investigated. The FSI-based model demonstrates strong potential for analyzing the impact of pore architecture on cell differentiation, although manufacturing challenges of hydrogel scaffolds may limit the practical application of these modification strategies.

**Supplementary Information:**

The online version contains supplementary material available at 10.1007/s10237-025-01976-1.

## Introduction

Hydrogels are three-dimensional (3D) crosslinked networks of hydrophilic polymers or macromers that swell in water, providing a supportive environment for cell proliferation and differentiation. Due to these properties, hydrogels are extensively used in cartilage tissue engineering (Hafezi et al. 2021). Furthermore, they are also effective bone graft substitutes for supporting the repair of large bone defects (Bush et al. 2016). Hydrogel scaffolds for tissue engineering applications should exhibit properties such as porosity, non-toxicity, favorable biocompatibility, support for cell differentiation, and promotion of new tissue regeneration (Ansari et al. 2024). In cartilage tissue engineering, hydrogel-based scaffolds provide an artificial microenvironment that emulates numerous characteristics of the native extracellular matrix (ECM) and exhibits responsiveness to various stimuli in a manner comparable to the natural ECM (Neves et al. 2020). Bioreactors or cultivation chambers are often used in cartilage tissue engineering as they enable in vitro tissue growth, deliver nutrients, and provide environmental signals to promote tissue development, thus stimulating tissue formation and regeneration (Castro et al. 2020b). In particular, hydrogel scaffolds are often mechanically stimulated, as this influences the phenotype and proliferation of the seeded cells (Schulz and Bader 2007; Meinert et al. 2017).

The influence of structural features such as pore size of scaffolds on stem cell differentiation can be examined in vitro, as demonstrated by Li et al. (2020), who studied the role of pore size in enhancing chondrogenic differentiation. However, in silico strategies offer a more cost-effective approach for studying the effects of scaffold pore design on mechanobiological forces and subsequent cell activities. Finite element (FE) analyses have been utilized to examine the influence of scaffold microstructural features, such as porosity, pore size, and pore shape, on mechanical properties, including elastic modulus, stiffness, strength, and stress concentration, as well as on biological activities (Olivares et al. 2009; Gómez et al. 2016; Du et al. 2019; Arjunan et al. 2020). Moreover, computational fluid dynamics (CFD) models have been employed to study how scaffold structural parameters affect the flow characteristics and, in particular, the resulting wall shear stress (WSS) in flow-through scaffolds, the permeability, as well as the biological performance of the scaffolds (Ali and Sen 2018; Zhianmanesh et al. 2019; Ouyang et al. 2019; Mahammod et al. 2020; Fallah et al. 2022).

Apart from FE analyses and CFD simulations, modeling a fluid–structure interaction (FSI) system offers researchers a more robust method to investigate the impact of scaffold pore architecture on cellular activities when subjected to mechanical stimuli from both fluid and solid environments. Fu et al. (2021) utilized FSI simulations to investigate the WSS on cell surfaces during deformation. Their study explored how variations in scaffold pore shape and size influence osteogenic differentiation. In the numerical study conducted by Zhao et al. (2020), a two-way FSI model was used to assess the impact of interstitial tissue formation on mechanical stimulation within a perfusion bioreactor. The study found that variations in cell morphology within the scaffold pores could lead to up to a ten-fold change in WSS. An FSI model can evaluate how changes in the microstructure of a regular scaffold, such as strand diameter and porosity, affect not only WSS and structural stresses and strains but also mass transport within the scaffold (Malvè et al. 2018). The application of FSI models in previous research primarily focused on enhancing scaffold architectures for bone tissue regeneration; moreover, a comprehensive analysis of cell differentiation across the entire scaffold surface, driven by dynamic mechanical stimulation, was not conducted (Zhao et al. 2016, 2020; Fu et al. 2021; Wang et al. 2022).

In this study, we employed a previously developed transient FSI modeling approach (Azizi et al. 2023) to enhance cartilage cell differentiation on the surface of a porous hydrogel scaffold by precisely adjusting its structural design. The geometric design parameters of the structured scaffold were modified, taking into account the manufacturability in using the direct ink writing (DIW) method. Mechanical stimulation was applied through a dynamic compression load with an amplitude of 5%, which exposed the seeded cells on the surface of the scaffold to mechanical stresses and strains, as well as compression-induced fluid wall shear stress. The mechanical and fluidic forces were analyzed in detail and discussed in relation to scaffold porosity, pore dimensions.

## Material and methods

The scaffold geometry, initially reported in Azizi et al. (2023), was modified in two steps to enhance cartilage cell differentiation under 5% compression amplitude, with the first modification step focusing on the variation of the uniform horizontal span (*Y*), and consequently the porosity of the regular scaffold described in the same study.

**Fig. 1:**
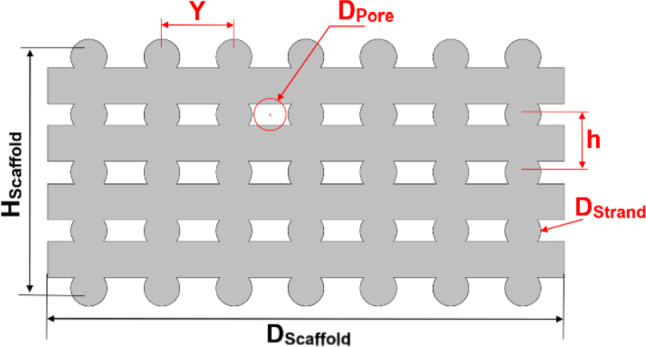
Sectional view of the scaffold. The parameters in black are fixed, while the parameters in red are variables in the modification process (The geometric parameters are detailed in Fig. S1 and Table S1 in Supplementary Material)

A sectional view of the original scaffold, displaying geometrical parameters, is presented in Fig. 1. The scaffold has a diameter (*D*_Scaffold_) of 10 mm, a height (*H*_Scaffold_) of 4.8 mm, and a strand diameter (*D*_Strand_) of 700 µm. The details of all geometrical parameters and their corresponding values for the original scaffold are reported in Fig. S1 and Table S1 in the Supplementary Material.

**Fig. 2:**
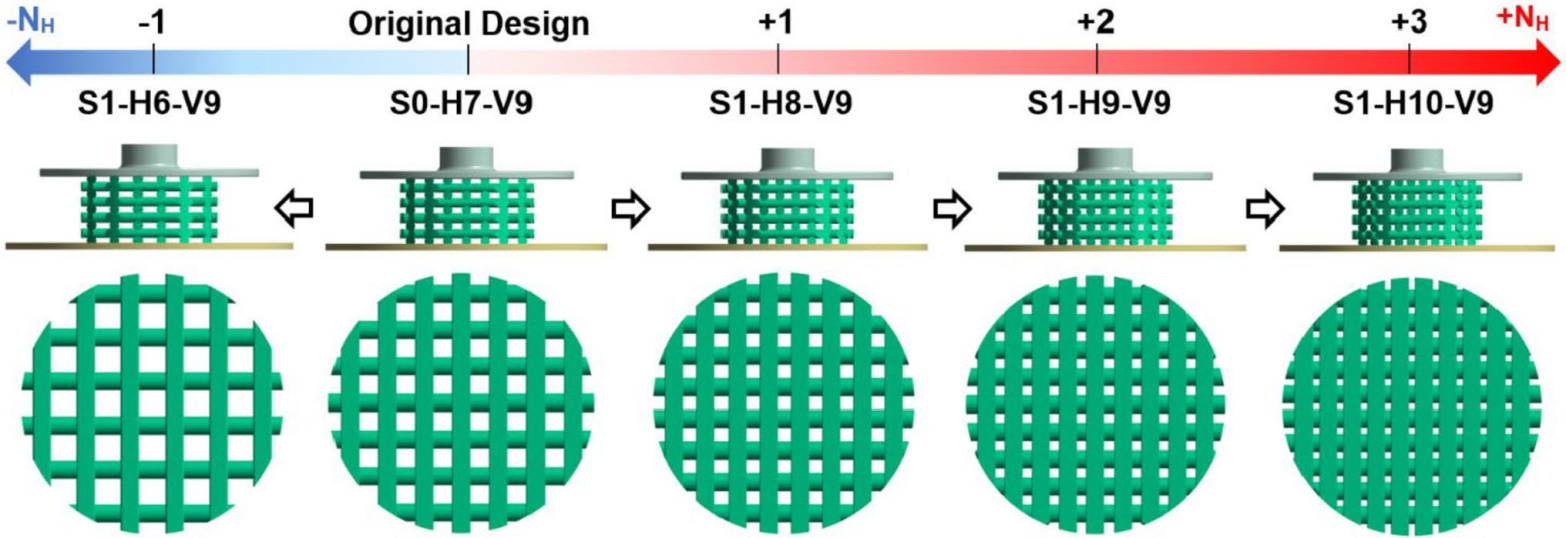
Step 1 of scaffold modification involves analyzing the impact of *Y* by varying *N*_*H*_

Based on Eq. S1 in the Supplementary Material, the horizontal span (*Y*) was modified by adding or removing strands to or from each horizontal layer, as shown in Fig. 2. This approach ensures that the diameter of the stands remains constant and the scaffolds can, therefore, still be produced using the direct ink writing (DIW) method. The various scaffold designs are denoted in this figure by using a combination of the letters *S*, *H*, and *V*. In this naming convention, *S* corresponds to the step number of scaffold modification (e.g., S1 = Step 1), H is followed by a number that specifies the number of strands in a horizontal layer (*N*_H_), and *V* is followed by the number of scaffold layers in the vertical direction (*N*_V_). For example, the scaffold S1-H6-V9 represents a scaffold modeled in step 1 with 6 strands per horizontal layer and 9 layers in the vertical direction. The original design described in Azizi et al. (2023) is designated as S0-H7-V9. Figure 2 clearly demonstrates that in step 1, the number of scaffold layers (V9) remains constant across all cases, whereas the number of horizontal stands varies from 6 to 10 (H6 to H10).

The porosity of the scaffolds was calculated as the ratio of the void volume to the total volume, expressed by the formula:

1 $$\text{Porosity}=1-\frac{{\text{Volume}}_{\text{ Scaffold}}}{{\text{Volume}}_{\text{ total}}}$$

To further evaluate the scaffold geometry, in addition to porosity, the parameter of minimal equivalent pore diameter (*D*_pore_) was also defined (Fig. 1). This symbolizes the diameter of an imaginary circle whose circular area corresponds to the minimum pore area formed by four strands within the scaffold, as shown in Fig. 1. *D*_pore_ can be calculated using the geometrical Eqs. S2–S3 in the Supplementary Material.

The design selected in step 1 (to be shown in the Sect. 3) was further modified in step 2. During this step, the scaffold porosity and outer dimensions (*D*_Scaffold_ and *H*_Scaffold_) were kept constant, while the number of scaffold layers (*N*_V_) was varied. This was achieved by scaling the scaffold design. Consequently, the geometric parameters adjusted in step 2 included *D*_Strand_, *D*_pore_, *Y*, and the vertical span (*h*). The objective of this modification was to examine the effect of *D*_pore_ on cell differentiation while maintaining consistent scaffold porosity and external dimensions. To achieve this, certain geometric constraints were managed to ensure the scaffold met the required structural design criteria. These constraints were established based on the ratios defined in Eqs. S4–S5 in the Supplementary Material. Considering these constants and the vertical geometric relationships, the value of *h* was calculated as a function of *N*_V_ (Eq. S6 in the Supplementary Material). In addition to *h*, the optimal value for *Y* should also be implemented in step 2 to finalize the geometric designs. A constant ratio was defined to utilize the optimal value for *Y* obtained from step 1 (Eq. S7 in the Supplementary Material). An overview of the strategy employed to modify the scaffolds in step 2 is summarized in a flowchart depicted in Fig. S2 in the Supplementary Material.

The details of the simulation setup for the FSI model, including the meshing strategy, boundary conditions of both FE and CFD models, material modeling, and the coupling strategy between fluid and solid environments, are thoroughly described in our previous work (Azizi et al. 2023). A brief summary of the simulation setup is also provided in this section.

The meshing strategy for this study was implemented using ANSYS Meshing 2020R2 (ANSYS Inc., PA, USA). A conformal mesh was applied between the solid and fluid zones, with a finer mesh specifically used for the scaffold surface. A mesh independence study was conducted on the original design S0-H7-V9 to determine the number of elements, as described in the Supplementary Material (Table S2 and Fig. S3).

**Fig. 3:**
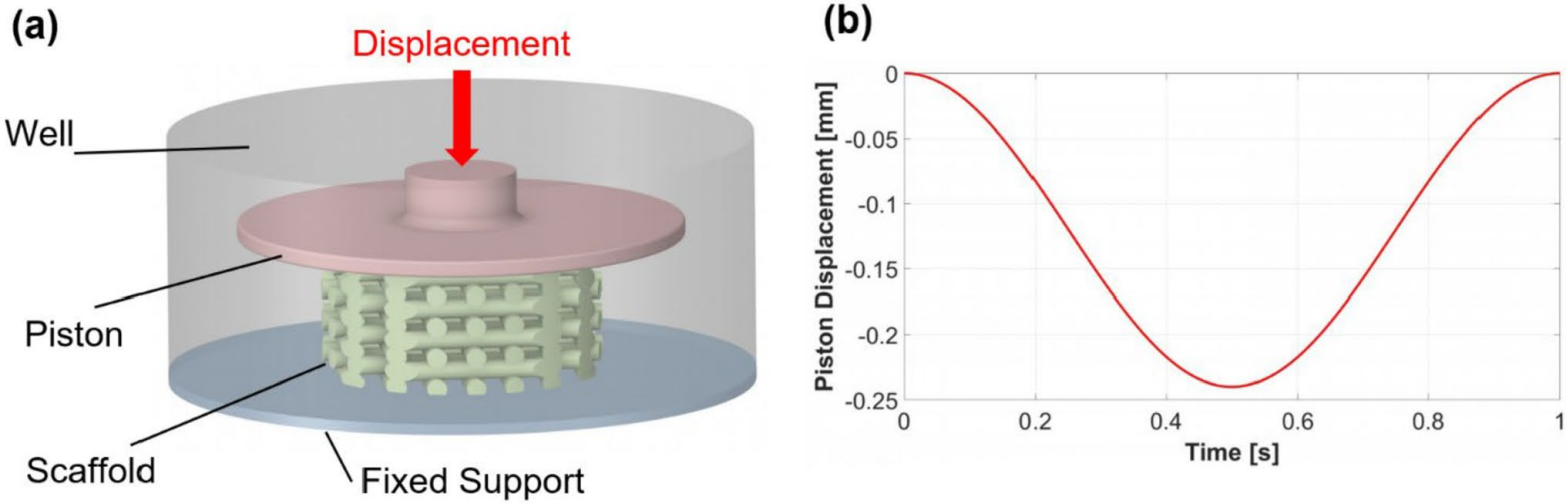
The FE model includes a piston that can move vertically, support at the bottom, and a scaffold between the two (**a**). Compressive load applied during a cycle with an amplitude of 5% (**b**)

The boundary conditions for the FE model were specified in ANSYS Transient Structural (Fig. 3). In this model (Fig. 3a), the piston is restricted to a vertical movement for compressing the scaffolds while the support remains fixed. The piston displacement followed a sinusoidal pattern with a frequency of 1 Hz and an amplitude equal to 5% of the scaffold height (Fig. 3b). The scaffold material was selected as pure oxidized alginate-gelatin (ADA-GEL) hydrogel, and its hyperelastic behavior was modeled using a one-term Ogden model. Hyperelastic materials are defined by a strain-energy density function (**W**) expressed in terms of strain or deformation tensors. The Lagrangian formulation for deriving strain and stress tensors is detailed in the ANSYS Mechanical APDL Theory Reference, Release 2020 R2. The general form of the Ogden model implemented in ANSYS is:

2 $${\varvec{W}}=\sum_{i=1}^{N}\frac{{\mu }_{i}}{{\alpha }_{i}}\left({\overline{{\lambda }_{1}}}^{{\alpha }_{i}}+ {\overline{{\lambda }_{2}}}^{{\alpha }_{i}}+ {\overline{{\lambda }_{3}}}^{{\alpha }_{i}}-3\right)+ \sum_{i=1}^{N}\frac{1}{{d}_{i}}{\left(J-1\right)}^{2i}$$

where **W** is the strain-energy potential, and is defined by the principal stretch ratios ${\lambda }_{1},{\lambda }_{2} \,\text{and }\,{\lambda }_{3}$, which quantify deformation, J is the volume ratio which defines the ratio of deformed to undeformed volume of the material, and ${\mu }_{i},{\alpha }_{i}\, \text{and}\, {d}_{i}$ are user-specified material constants. The material constants for the Ogden model were determined from compression-tension measurements (Distler et al. 2021), with *μ*_1_ =  − 5.8 kPa and *α*_1_ =  − 1.3, under the assumption of fully incompressible material behavior (*d*_1_ = 0). All simulations were performed for a single loading cycle, as the viscoelastic properties of the scaffold were not considered, making simulations for successive cycles inapplicable.

Fluid flow within and around the scaffolds was simulated using ANSYS Fluent. A dynamic mesh approach was applied to specific fluid regions that adjusted to the movements transmitted from the solid zones (i.e., piston and scaffold). The CFD model boundary conditions included fluid–solid interface zones for the coupled solid and fluid regions, a pressure outlet at the top surface of the well, and no-slip walls at the well's bottom and cylindrical walls. The culture medium was modeled as a Newtonian, incompressible, and laminar flow, with a density of 1000 kg/m^3^ and a dynamic viscosity of $1.45\times {10}^{-3}$ Pa s. The maximum Reynolds number (Re) based on *D*_Strand_ was approximately 1.5 in step 1 and around 1.6 in step 2, both calculated at the simulation time corresponding to cell differentiation prediction.

The FSI system was a 3D, transient, and one-way model. The two solid and fluid environments were coupled based on a co-simulation strategy using ANSYS System Coupling. Since the fluid flow was induced from the solid motion, and on the other hand, the fluid motion did not have a significant influence on mechanical movements, the one-way approach was applied. To assess the accuracy of our transient model, a sensitivity study on the “time-step size” was conducted. The results of this study can be found in the Supplementary Material where we evaluated the influence of three different time-step sizes: large, medium, and small (Fig. S4 in the Supplementary Material). It was observed that cell differentiation was not significantly dependent on the chosen time steps. Therefore, to perform the simulations faster, a large time-step size of 0.01 s was selected.

Prendergast et al. (1997) developed a mechanoregulation theory proposing that the fate of mesenchymal stem cells (MSCs) is governed by mechanical strain and fluid flow. A modified version of this mechanoregulation theory was utilized to calculate the stimuli and thereby determine the resulting cell phenotype (Olivares et al. 2009; Sandino and Lacroix 2011; Hendrikson et al. 2017; Azizi et al. 2023):

3 $$S= \frac{\text{OSS}}{a}+\frac{\text{WSS}}{b}$$

where *S* is the stimuli, OSS is the octahedral shear strain, WSS is wall shear stress, and *a* and *b* are constants equal to 0.0375 and 10 mPa. The cells’ phenotype was classified based on the *S* value (Olivares et al. 2009; Sandino and Lacroix 2011; Hendrikson et al. 2017; Azizi et al. 2023), as outlined in Table 1. The OSS in Eq. 3 was computed from the FE model using the following equation:

4 $$\text{OSS} = \frac{2}{3} \sqrt{{{(\varepsilon }_{1}-{\varepsilon }_{2})}^{2}+{{(\varepsilon }_{2}-{\varepsilon }_{3})}^{2}+{{(\varepsilon }_{3}-{\varepsilon }_{1})}^{2} }$$

where *ε*_1_, *ε*_2_, and *ε*_3_ are the elastic principal strains. On the other hand, the WSS in Eq. 3 was computed on the surface of the scaffolds from the CFD model as follows:

5 $$\text{WSS}=\mu \frac{\partial {\varvec{u}}}{\partial {\varvec{n}}}$$

where $\mu$ is the dynamic viscosity, and $\frac{\partial {\varvec{u}}}{\partial {\varvec{n}}}$ denotes the normal velocity gradient of the fluid on the wall.

**Table 1:** Predicting tissue phenotypes based on computed stimuli values

$S\le 0.01$	$0.01<S\le 1$	$1<S\le 3$	$3<S\le 6$	$S>6$
Very low stimuli	Bone tissue differentiation	Cartilage tissue differentiation	Fibrous tissue differentiation	Very high stimuli

Utilizing a conformal meshing approach at the interface of the FSI model ensured that both FE and CFD elements shared identical external nodes. Consequently, OSS and WSS values could be computed at these nodes to determine the *S* value. The outcomes of FE and CFD simulations were matched at shared nodes employing a MATLAB script created for calculating *S*.

Given the transient nature of the simulations, mechanical and fluidic forces varied at each simulation time point. The methodology for determining the time point at which cell differentiation occurred is described in our previous study (Azizi et al. 2023).

## Results

### Step 1: variation of *Y*

As discussed earlier, the impact of altering *Y* on cell differentiation was investigated in step 1. The geometric properties of the scaffolds from step 1 (Fig. 2) are outlined in Table 2, with *N*_v_, *D*_strand_ and *h* held constant, while *Y*, *D*_pore_, and porosity decrease with increasing *N*_H_.

**Table 2:**
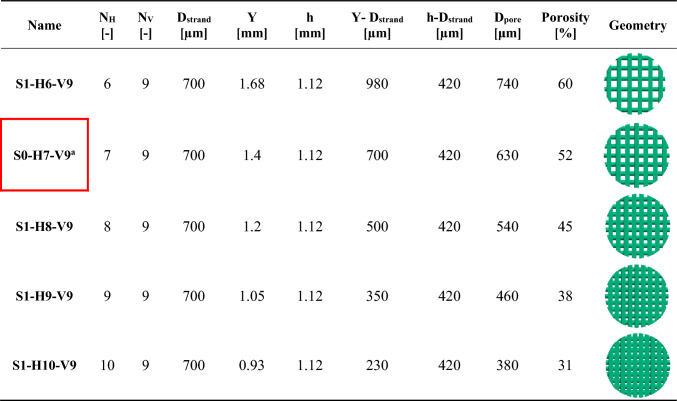
Details of the geometric modification in step 1. The geometric parameters are depicted in Fig. 1

^**a**^Original design

**Fig. 4:**
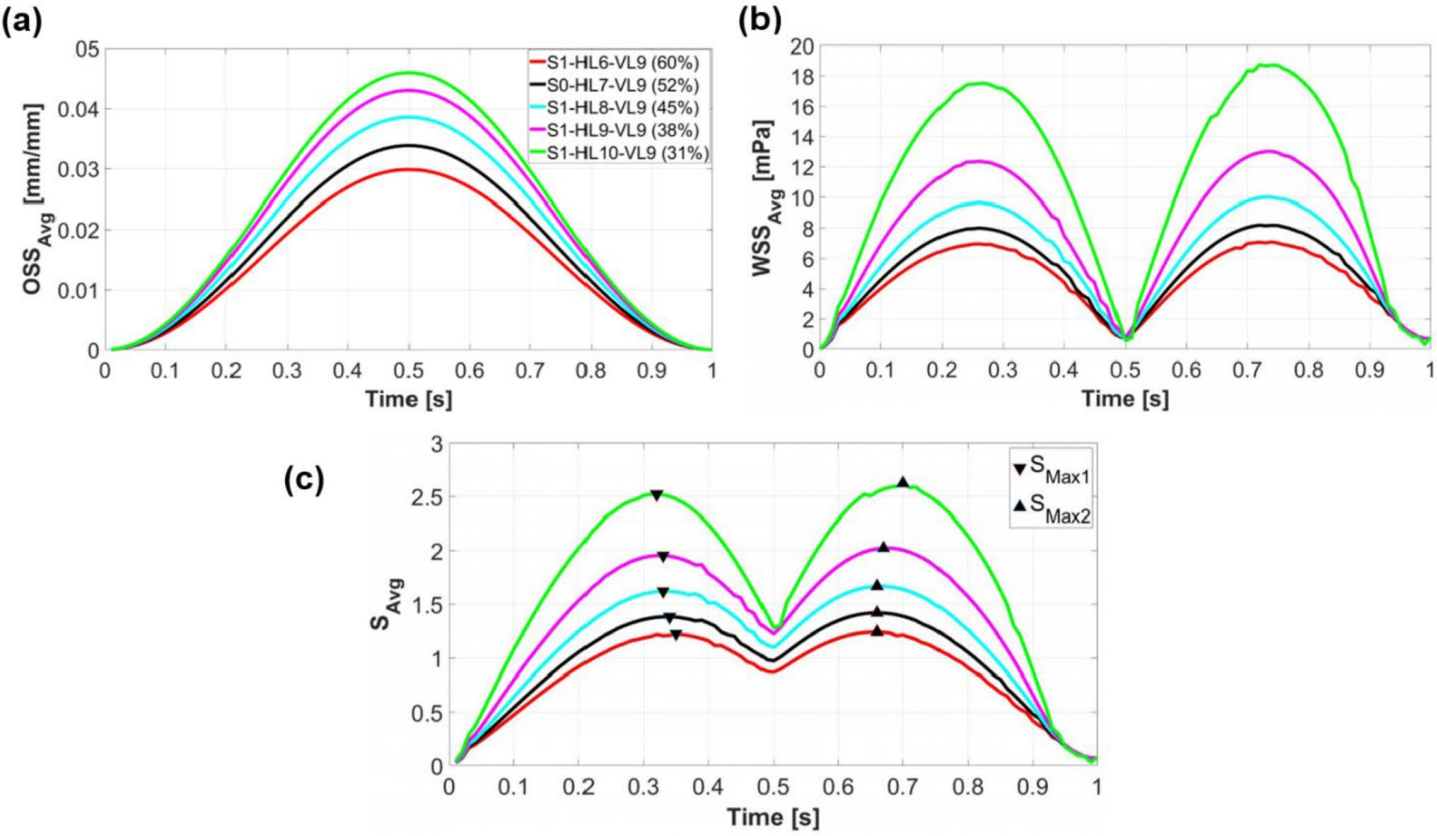
$\text{OSS}_{\rm avg}$(**a**), $\text{WSS}_{\rm avg}$ (**b**), and ${S}_{\rm avg}$ as a function of $\text{OSS}_{\rm avg}$ and $\text{WSS}_{\rm avg}$ (**c**) during a loading cycle for various scaffold designs in step 1. The results of step 1 are compared with those of the original scaffold S0-H7-V9 (black curves). The triangular markers indicate the maximum values of ${S}_{\rm avg}$ when the piston moves downwards (${S}_{\rm Max1}$) and upwards (${S}_{\rm Max2}$)

At each simulation time, the average stimulus value (*S*_avg_​) is calculated using Eq. 2. This value is a function of the area-weighted average of wall shear stress across the entire scaffold surface (WSS_avg_) and the arithmetic mean of the octahedral shear strain from all FE nodes on the scaffold surface (OSS_avg_). Figure 4 illustrates the change of OSS_avg_, WSS_avg_, and *S*_avg_ during a loading cycle for different scaffold designs in step 1. Both OSS_avg_ (Fig. 4a) and WSS_avg_ (Fig. 4b) exhibit a rise with increasing *N*_H_ (or decreasing porosity). The maximum value of OSS_avg_ for all designs occurred at $t=0.5s$, corresponding to the piston’s lowest position, as displayed in Fig. 4a (Piston displacement curve in Fig. 3b helps identify the piston’s position at each simulation time). OSS_avg_ reached 0.03 for scaffold S1-H6-V9 and increased to 0.046 for scaffold S1-H10-V9. On the other hand, maximum values of WSS_avg_ occurred when the piston was returning to its original position (Fig. 4b). WSS_avg_ ranged from 0 to 7.09 mPa for scaffold S1-H6-V9 and reached a range of 0 to 18.74 mPa for scaffold S1-H10-V9. Based on Eq. 3, as OSS_avg_ and WSS_avg_ increase, *S*_avg_ also grows, as can be seen in Fig. 4c. The two maxima of *S*_avg_ curves, *S*_Max1_ and *S*_Max2_, are also displayed in Fig. 4c. *S*_Max1_ corresponds to the time when *S*_avg_ reaches its maximum during scaffold compression ($0\le t<0.5s$), while *S*_Max2_ represents the time point when *S*_avg_ maximized during the scaffold release phase ($0.5s\le t<1s$).

**Fig. 5:**
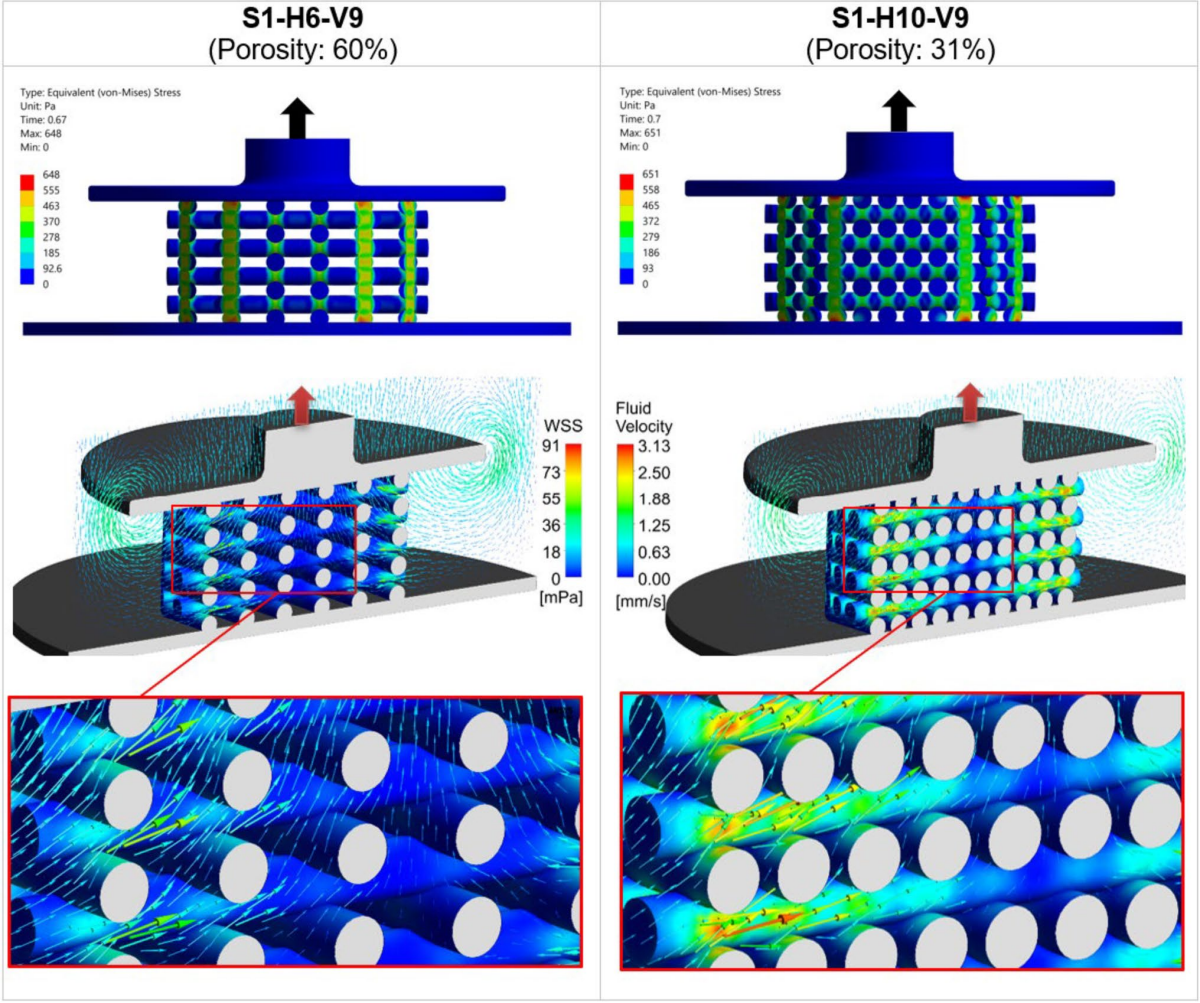
The left column displays the mechanical deformation and fluid flow in the scaffold S1-H6-V9 (60% porosity), while the right column presents those of S1-H10-V9 (31% porosity). The top section shows the distribution of von Mises stress, and the middle section displays WSS with fluid velocity vectors, with detailed views of scaffolds below. The color scales for WSS and fluid velocity are consistent across both cases for direct comparison purposes. All results are shown at the time point when the average stimulus (${S}_{\rm Avg}$) reaches its maximum value during the piston’s upward movement (${S}_{\rm Max2}$ in Fig. 4c)

Figure 5 illustrates the influence of scaffold porosity on the mechanical stress and fluid flow inside the scaffold by comparing two scaffold designs from step 1: S1-H6-V9 with 60% porosity (the largest porosity) and S1-H10-V9 with 31% porosity (the smallest porosity). These post-processing results were captured at the simulation time point when ${S}_{\rm Max2}$ occurred, during the piston release phase, as indicated by the upward arrows on the top of the piston. At the top of Fig. 5, a comparison of the von Mises stress between the two scaffolds is shown, highlighting that stress concentrations are higher at the intersections of the scaffold strands. In the middle section of Fig. 5, the WSS contours on the scaffold surfaces are displayed alongside fluid velocity vectors, with detailed views below. The color scales for WSS and fluid velocity are kept uniform for both scaffolds to allow for easier comparison. The figure clearly demonstrates that both the WSS and the developed velocity field are greater for the scaffold with smaller porosity (S1-H10-V9). Additionally, it is evident that the inner regions of low WSS within the scaffold decrease as porosity decreases (Fig. 5).

**Fig. 6:**
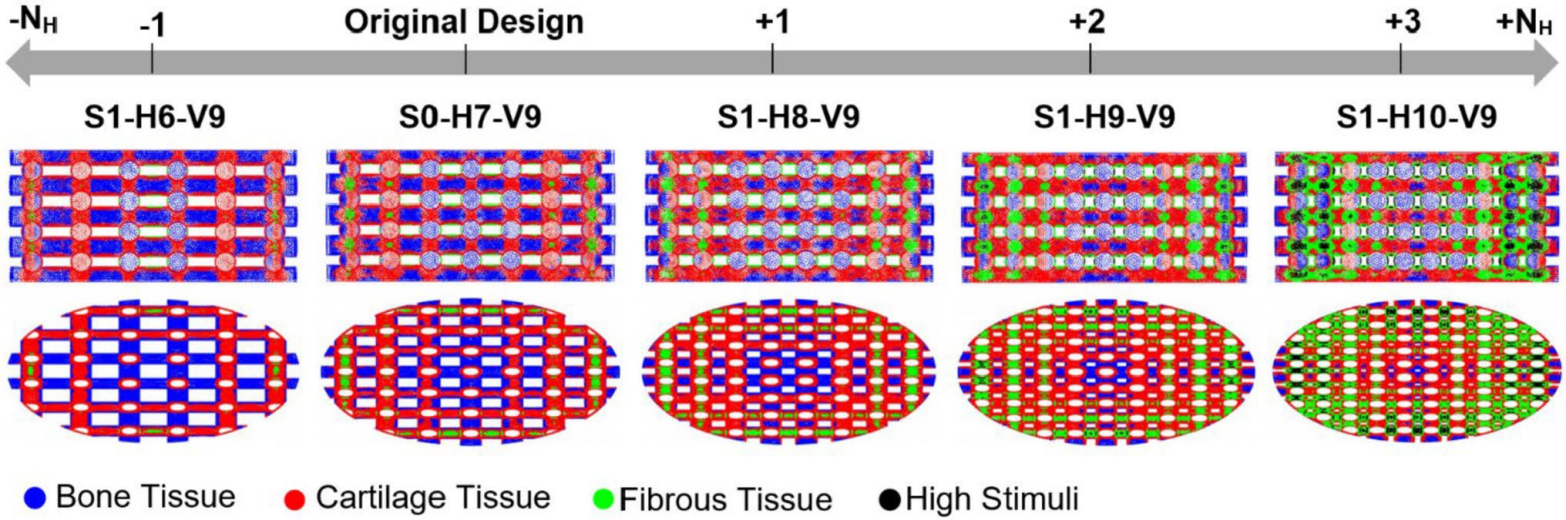
The effect of altering the geometric parameter *Y* on cell differentiation (step 1). *Y* was changed by adding or removing strands from each horizontal layer of the original scaffold (*S0-H7-V9*). Each color on the scaffold surface represents a distinct cell phenotype

The impact of the parameter *Y* (or porosity) on cell differentiation is depicted in Fig. 6. Removing one strand from each horizontal scaffold layer increased bone tissue differentiation, as shown in Fig. 6. In contrast, adding strands to each horizontal layer predicted a reduction in bone cell differentiation and growth of both cartilage and fibrous cell differentiation (Fig. 6). The figure illustrates that as we progress along the depicted axis from the original design toward the right endpoint (S1-H10-V9), the central scaffold regions become less supportive of bone cell differentiation. In contrast, the peripheral regions experience increased mechanical stimulation, which promotes fibrous tissue differentiation or leads to cell death.

**Fig. 7:**
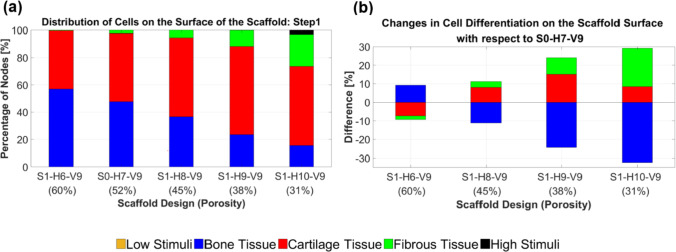
An overview of the percentage of differentiated cells on the surface of the scaffolds in step 1 (**a**). The changes in cell differentiation observed in each structural design relative to the original scaffold (**b**) indicate that scaffold S1-H9-V9 led to improved cartilage cell differentiation

Based on the graphs in Fig. 7, it is evident which scaffold design enhanced cartilage cell differentiation in step 1. Figure 7a provides an overview of the percentage of differentiated cells across the entire surface of the designed scaffolds. Moreover, Fig. 7b quantitatively illustrates how cell differentiation varies for each cell phenotype based on scaffold structural design. The optimal design for cartilage cell differentiation in step 1 involves adding two strands to each horizontal layer (i.e., S1-H9-V9), as shown in Fig. 7b. This scaffold design enhances cartilage cell differentiation by 15.01% and reduces bone cell differentiation by 24.34% compared to the original design (S0-H7-V9).

### Step 2: variation of ***h*** and ***D***_strand_

**Fig. 8:**
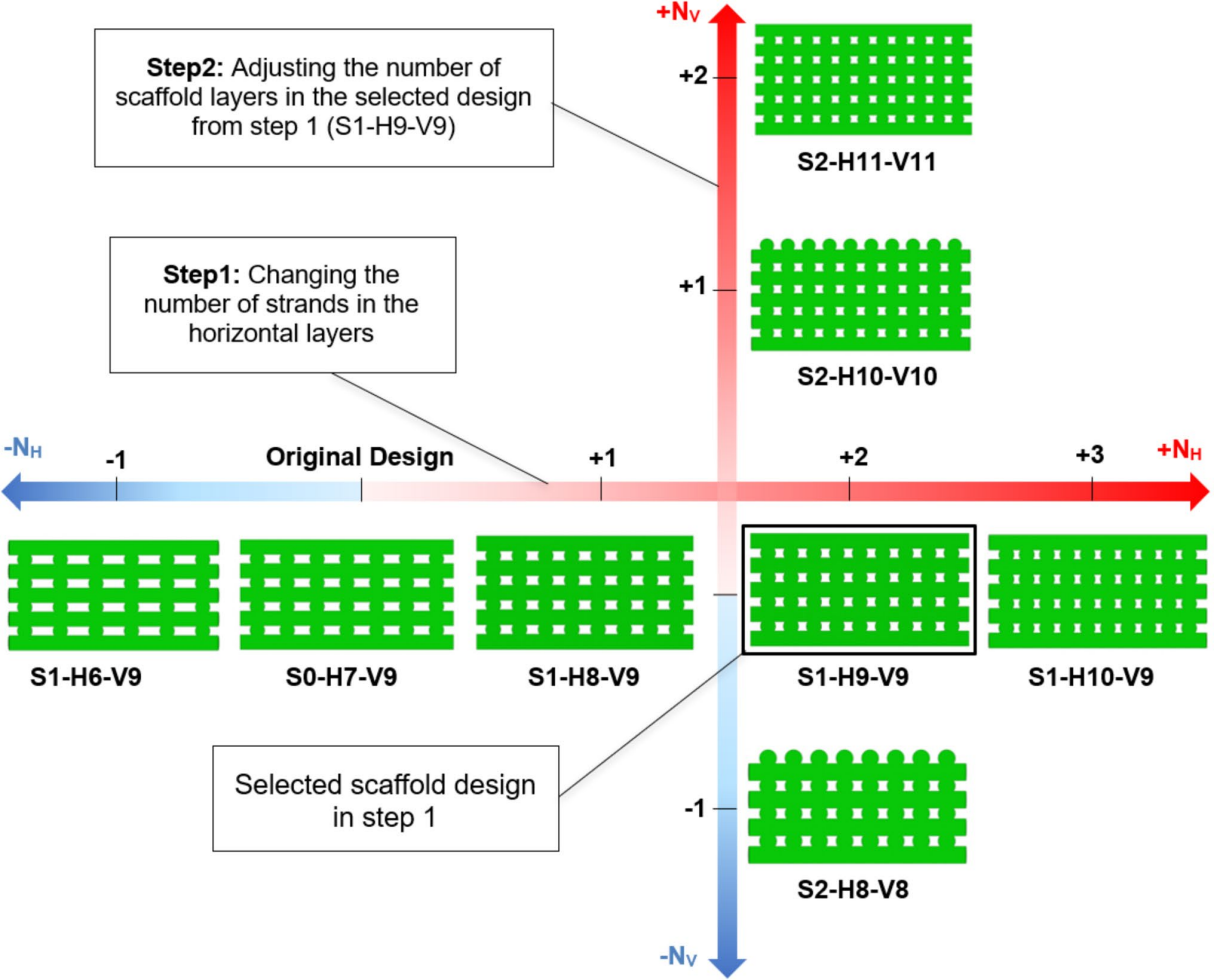
The two-step modification of the scaffold S0-H7-V9

The optimal scaffold design from step 1, S1-H9-V9, was selected for further modification in step 2. As outlined in Sect. 2, the approach in step 2 involved adding or removing scaffold layers by scaling the scaffold S1-H9-V9 while maintaining constant porosity and outer dimensions (*D*_Scaffold_​ and *H*_Scaffold_). To meet the modification design criteria, specifically adjusting *N*_V_ while keeping *D*_Scaffold_ and *H*_Scaffold_ unchanged, this scaling process affected not only *h* but also *D*_Strand_, *Y*, *N*_V_ and consequently, *D*_pore_. Scaling also considered the scaffolds’ ability to be manufactured using DIW. The primary focus of this step was to examine the influence of *D*_pore_ on cell differentiation and to compare this effect with the influence of porosity observed in step 1. Detailed methodology for the modification process in step 2 is provided in the Supplementary Material. Figure 8 displays the applied strategy for the two-step modification process of the original scaffold S0-H7-V9.

The values of the geometrical parameters for step 2 are presented in Table 3, and the geometric properties of scaffold S1-H9-V9 are also included in this table as a reference for comparison. It can be observed from Table 3 that, although the porosity of the scaffolds did not change significantly, the parameter *D*_pore_ varies proportionally with the alteration of scaffold layers. The constant ${x}_{3}$ (Eq. S7 in the Supplementary Material) was crucial in maintaining equal values for *N*_H_ and *N*_V_.

**Table 3:**
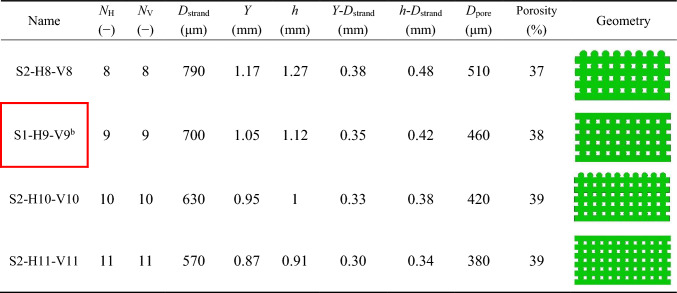
Geometry details of design optimizations in step 2

^**b**^**Selected from step 1.**

**Fig. 9:**
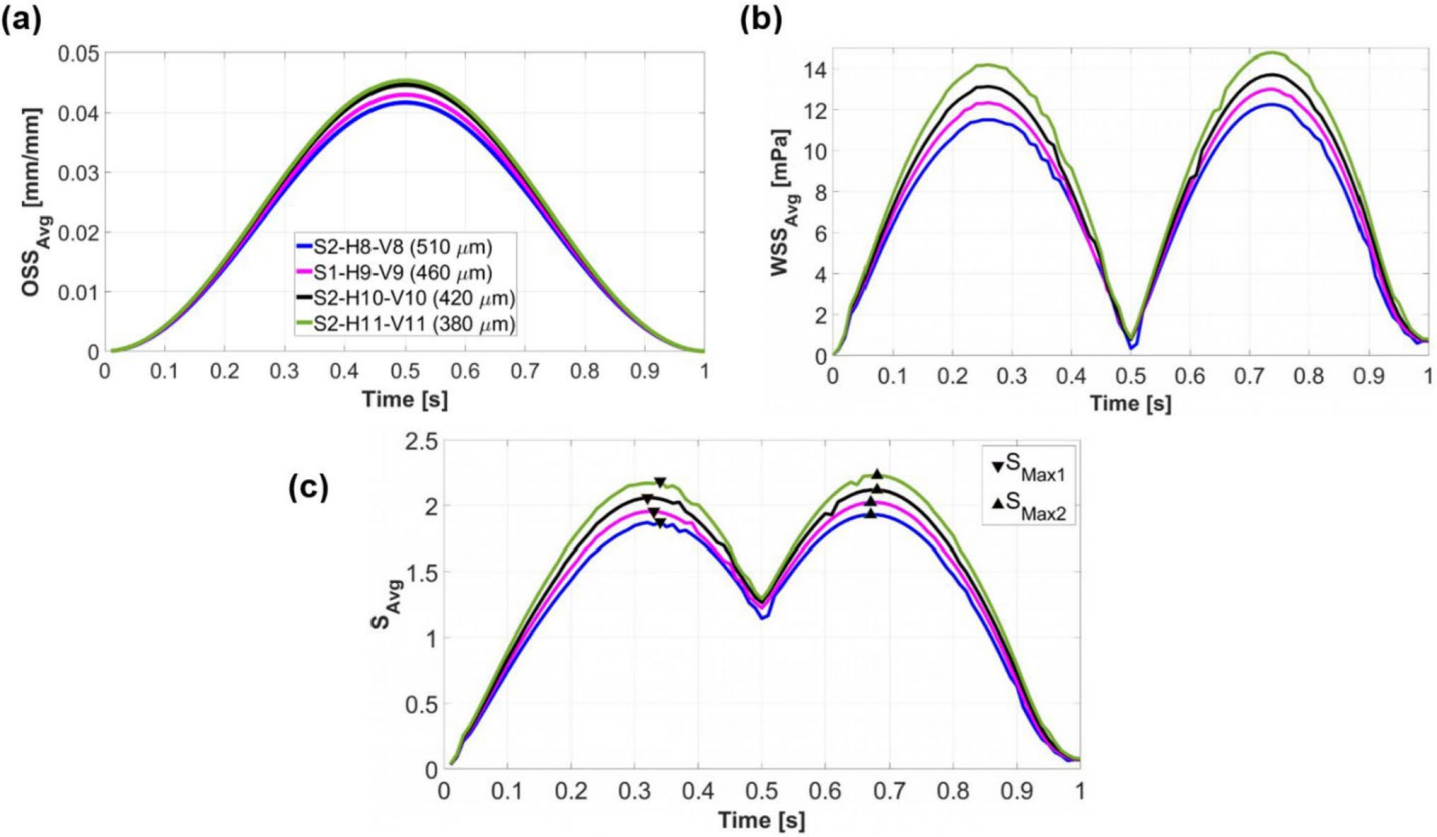
$\text{OSS}_{\rm avg}$(**a**), $\text{WSS}_{\rm avg}$ (**b**), and ${S}_{\rm avg}$ as a function of $\text{OSS}_{\rm avg}$ and $\text{WSS}_{\rm avg}$ (**c**) throughout a loading cycle for various scaffold designs in step 2. The legend in (**a**) determines the scaffold architectures with their corresponding *D*_*pore*_. Triangular markers (**c**) exhibit the maximum values of ${S}_{\rm avg}$ during downwards (${S}_{\rm Max1}$) and upward (${S}_{\rm Max2}$) piston movements

Figure 9a–b illustrates that vertically increasing the number of scaffold layers (decreasing *D*_pore_) resulted in higher OSS_avg_ and WSS_avg_ values. Consequently, *S*_avg_ exhibits a similar trend to OSS_avg_ and WSS_avg_, with the highest values observed for the scaffold design S2-H11-V11 (Fig. 9c). The time points associated with *S*_Max2_ were considered for predicting cell differentiation across the entire scaffold surface. The change in OSS_avg_ was not significant, as shown in Fig. 9a. The scaffold with the largest *D*_pore_ (S2-H8-V8) reached a maximum OSS_avg_ value of 0.042, whereas the scaffold with the smallest *D*_pore_ (S2-H11-V11) reached a maximum OSS_avg_ value of 0.045, representing only a 7% increase. In contrast, variations in WSS_avg_ were more pronounced, as illustrated in Fig. 9b. The scaffold S2-H8-V8 achieved a maximum WSS_avg_ value of 12.26 mPa, while S2-H11-V11 reached a maximum value of 14.76 mPa, corresponding to approximately a 20% increase.

**Fig. 10:**
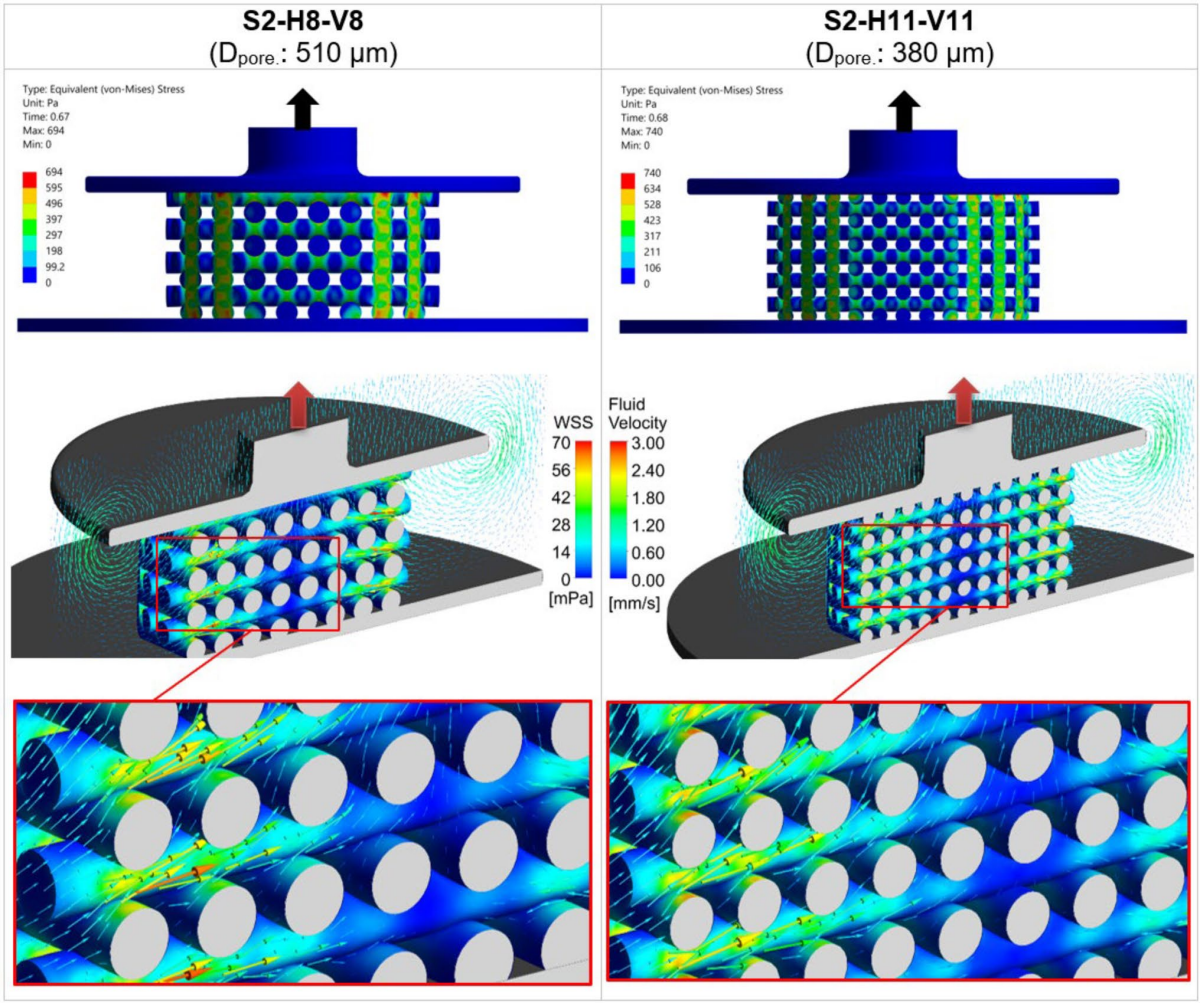
The left column displays the mechanical and fluidic properties of the scaffold S2-H8-V8 (*D*_pore_: 510 μm), while the right column presents those of S2-H11-V11 (*D*_pore_: 380 μm). The top section shows the distribution of von Mises stress, and the middle section displays wall shear stress (WSS) with fluid velocity vectors, accompanied by detailed views of scaffolds below. The color scales for WSS and fluid velocity are consistent across both cases for direct comparison. All results are shown at the time point when the average stimulus (${S}_{\rm Avg}$) reaches its maximum value during the piston’s upward movement (${S}_{\rm Max2}$ in Fig. 10c)

Figure 10 compares the mechanical stress and fluid flow in scaffold architectures S2-H8-V8 and S2-H11-V11 in step 2. At the top of the figure, the von Mises stress of these scaffolds at the simulation times of maximum *S*_avg_ is compared. In the middle section, the WSS contours of these scaffolds are depicted alongside fluid velocity vectors, with detailed views of each scaffold below. As with Fig. 5, the color scales for both WSS and fluid velocity remain consistent between the two scaffolds to facilitate a comparison. The detailed view indicates that WSS is slightly greater for scaffold S2-H11-V11 compared to S2-H8-V8, although there is no significant difference in fluid velocity vectors between the two scaffolds.

**Fig. 11:**
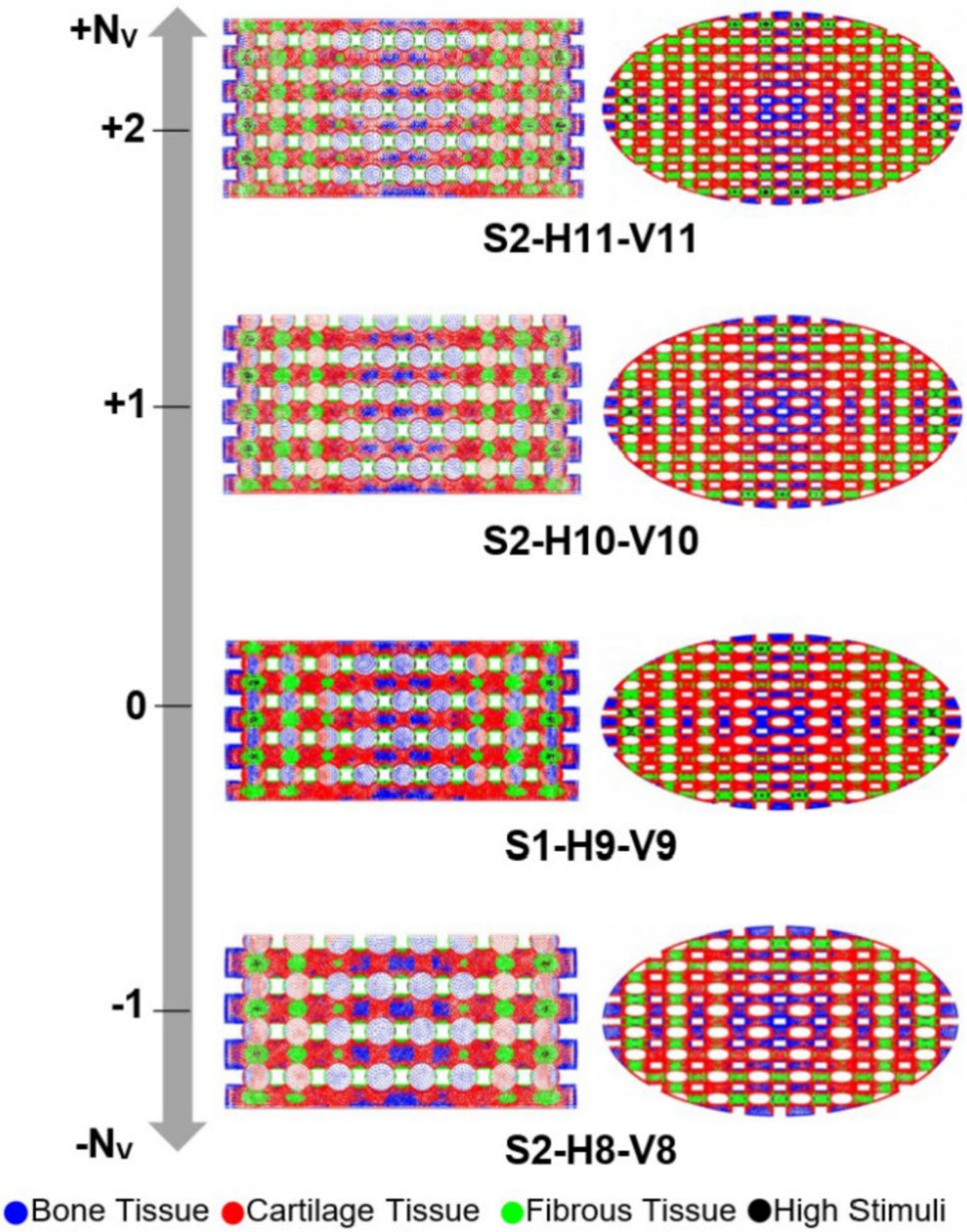
Influence of scaling the selected scaffold in step 1 (S1-H9-V9) by adding or removing horizontal layers from it. Each color on the scaffold surface represents a distinct cell phenotype

**Fig. 12:**
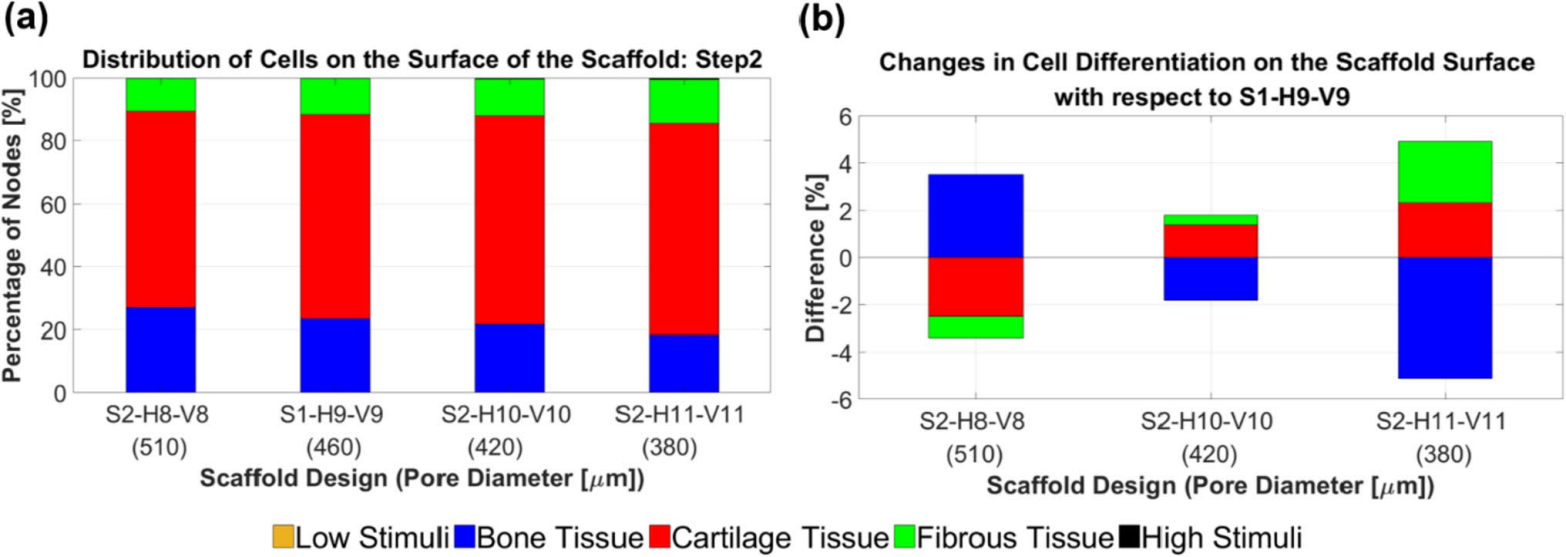
An Analysis of the percentage of differentiated cells on the surface of the scaffolds in step 2 (**a**). The changes in cell differentiation relative to the selected design in step 1 (**b**) indicate that scaffold designs S2-H10-V10 and S2-H11-V11 resulted in increased differentiation of cartilage cells

The distribution of cell phenotypes on the designed scaffolds in step 2 is presented in Fig. 11. However, from this figure, it is not immediately clear which structural design improved cartilage cell differentiation. Hence, a quantitative analysis of cell differentiation on the surface of these scaffolds elucidated the results (Fig. 12).

Figure 12 depicts the impact of changing the number of horizontal layers (according to the strategy outlined in step 2) on cell differentiation across the entire scaffold surface. Scaffold design S2-H11-V11 demonstrated an improvement of 2.32% in cartilage cell differentiation and a reduction of 5.13% in bone cell differentiation compared to scaffold S1-H9-V9, as shown in Fig. 12b. Since the difference in cartilage cell differentiation improvement between the S2-H10-V10 and S2-H11-V11 models was insignificant (< 1%), the modification process in step 2 was concluded with scaffold S2-H11-V11. In other words, it is anticipated that adding more layers to scaffold S2-H11-V11 based on the applied strategy in step 2 would not further enhance cartilage cell differentiation.

## Discussion

In this research, we adopted an FSI-based modeling approach to investigate the effect of scaffold architectures on their mechanobiological properties under mechanical stimulation. Our results show that cartilage cell differentiation can be enhanced on the surface of structured scaffolds through geometric modifications. The compression amplitude was set to 5% to investigate the influence of changing scaffold structures on cartilage cell differentiation. This specific compression amplitude was chosen because it resulted in nearly equal percentages of differentiated bone and cartilage cells on the surface of the primary scaffold, as reported by Azizi et al. (2023). This balance facilitated observing how structural modifications, such as changes in porosity or pore size, could shift differentiation toward more bone or cartilage cells. Furthermore, our selected 5% compression is within the mid-range of compression amplitudes, which has been shown to be promising in previous in vitro studies. These studies suggest that compressions of up to 10% can enhance the biochemical and biomechanical properties of scaffolds (El-Ayoubi et al. 2011; Shahin and Doran 2012; Nebelung et al. 2012; Salinas et al. 2018).

### Step 1: Impact of porosity

#### Compressive mechanical stimulation

The stiffness of scaffolds increases with decreasing porosity (Zhao et al. 2021). In step 1 of our study, this means that applying the same compression amplitude to a scaffold with lower porosity (and thus higher overall structural stiffness) requires greater mechanical force. Since the displacement of the piston as a boundary condition is the same for all simulations, the overall structural stiffnesses of the scaffold do not affect the mechanical compression process. However, compressive mechanical stimulation is influenced by two other factors. On the one hand, the time *S*_Max_ is different for all scaffolds, so at the respective time *S*_Max_, there is a different degree of compression. The higher the compression at time *S*_Max_, the stronger the mechanical stimulation. As the *S*_Max_ time points do not vary so much for all results, the compression at the *S*_Max_ time point is almost similar for all simulations. Secondly, the different scaffold designs have a different number of strand intersections, with a larger number of intersections resulting in a higher overall mechanical stimulation. This parameter varies significantly for all designs, meaning that this parameter has a dominant influence on compressive mechanical stimulation. This effect is evident in Fig. 4a, where OSS_avg_ reached its highest values for the scaffold with the lowest porosity and consequently the highest number of intersections, specifically S1-H10-V9, in step 1. The impact of porosity on the mechanical properties of the scaffolds is further illustrated in Fig. 5, where an increase in the number of strands in each layer led to more strand intersections and, consequently, more regions on the scaffold exhibiting high von Mises stress values. This resulted in a higher overall OSS for scaffolds with reduced porosity. This trend is consistent with findings reported by Hendrikson et al. (2017). For instance, their study showed that a scaffold architecture with a 0/45 pattern and 72.6% porosity exhibited an OSS_avg_ of 0.035, while a 0/45 offset architecture with 63.6% porosity achieved an OSS_avg_ of 0.053.

#### Fluid-induced shear stimulation

In a study by Ali and Sen (2018), scaffolds with lattice-based and gyroid architectures were designed with six different porosity levels to investigate and compare their permeability and fluid-induced WSS. They found that decreasing the porosity of lattice-based scaffolds from 90 to 65% led to an increase in the average WSS from 23.5 to 48.6 mPa for Newtonian fluids, effectively doubling the WSS_avg_ with a 25% reduction in porosity. Our results show that a 29% decrease in porosity (from 60 to 31%) in step 1 resulted in a 2.6 times increase in the maximum values of WSS_avg_, as seen in Fig. 4b. The results of both studies show a similar trend, even though the findings are not fully comparable. This can be attributed to the fact that Ali and Sen (2018) calculated fluid-induced WSS, not WSS due to compression-induced flow, and also to differences in the geometrical designs. Zhao et al. (2016) also demonstrated that lower porosity results in higher WSS under dynamic compression. For instance, for a scaffold design with cubic pores, reducing the porosity by 30% (from 90 to 60%) led to a 1.4 times increase in WSS_avg_ (from 2.5 to 3.5 mPa) under 1% compression (Zhao et al. 2016, 2021). Given that the boundary conditions remained consistent across all simulations in step 1, reducing the pore dimension inside the scaffold resulted in an increase in compression-induced velocity (Fig. 5). This prompts the question of how fluid velocity magnitude relates to WSS. Analytically, using the Blasius equation for flat plates, it is shown that WSS is proportional to the external velocity raised to the power of 1.5 (White Frank 1998). Thus, it can be inferred that WSS inside the scaffold increases with increasing velocity. However, obtaining an analytical solution for the relationship between WSS and fluid velocity magnitude in general three-dimensional (3D) bodies is not feasible without simplifications (Schlichting and Gersten 2017). Consequently, CFD serves as a valuable tool for WSS based on factors such as fluid velocity, porosity, and pore size.

#### Cell differentiation

The control of scaffold porosity to induce cartilage cell differentiation under 5% compression was examined in step 1 (Fig. 6). Castro et al. (2020a) used an FE model to investigate the influence of porosity in three different scaffolds under various loading conditions on mechanobiological outcomes. For a 6% compression applied for 30 s, their FE model of Schwartz P (SP) scaffolds with porosities of 60%, 70%, and 80% resulted in 58.87%, 62.50%, and 66.85% bone formation, respectively (Castro et al. 2020a). Conversely, cartilage differentiation for the same scaffolds was 32.84%, 30.73%, and 27.98%, respectively (Castro et al. 2020a). These findings align with the bone differentiation trends observed in step 1 of our study. However, in our research, cartilage differentiation initially increased as porosity rose from 31 to 38% and then decreased as porosity further increased from 38 to 60%.

Porosity also plays a crucial role in bone tissue engineering. Previous studies have generally selected porosity values between 50 and 80% for bone scaffolds (Foroughi et al. 2025). However, excessive porosity can weaken the scaffold structurally and increase permeability, potentially leading to cell washout (Wang et al. 2017). These findings align with our results for bone cell differentiation in step 1, where scaffolds S0-H7-V9 and S1-H6-V9, with porosities of 52% and 60%, respectively, demonstrated superior performance compared to other scaffolds (Fig. 7).

To evaluate the contribution of WSS and OSS to the maximum mechanical stimulus (${S}_{\text{Max}2}$) during a loading cycle, their respective portions were analyzed. For the scaffold with the highest porosity (S1-H6-V9), WSS accounted for 51.15%, while OSS contributed 48.85% of the total stimulus. In contrast, for the scaffold with the lowest porosity (S1-H10-V9), WSS represented 69.93%, whereas OSS accounted for 30.07% of the stimulus. These results indicate that as porosity decreases, mechanical stimulation due to fluid shear stress becomes more dominant compared to structural strain-induced stimulation.

### Step 2: impact of pore diameter ***D***_pore_ (constant porosity)

The modified scaffold from step 1, namely S1-H9-V9, was scaled in step 2 while keeping the porosity and outer dimensions of the scaffold constant. This step involved evaluating the influence of pore diameter (*D*_pore_) on cell phenotype prediction and comparing it to the effect of porosity. After scaling, *D*_Strand_ ranged from 570 to 790 µm, ensuring that the scaffolds remained manufacturable using the DIW process. Both OSS_avg_ and WSS_avg_ were predicted to be higher for designs with smaller pore diameters (Fig. 9a–b). Additionally, Fig. 12b shows that scaffolds with larger pore diameters favor bone cell differentiation and relatively less cartilage differentiation. Fu et al. (2021) showed in their numerical study that the maximum values of WSS on the scaffold surfaces decreased as the pore size increased. Moreover, their simulations confirmed that mechanical stimulation led to more significant osteogenic differentiation for larger pore sizes (Fu et al. 2021). In the research study by Bartnikowski et al. (2014), WSS was computed using CFD for scaffold architectural variations, maintaining a constant porosity. The trend of WSS variation as a function of pore size observed in their study corresponds with our findings. Unlike in step 1, the fluid velocity in step 2 did not change significantly with the reduction in pore size, since porosity remained nearly constant (Fig. 10). The rise in WSS_avg_ for smaller pore sizes (Fig. 9b) can be attributed to the diameter of the strands. As the strand diameter decreased (with a corresponding reduction in *D*_pore_) in step 2, the surface curvature increased, leading to sharper velocity gradients and, consequently, higher WSS values (Okechi et al. 2017). Additionally, reducing pore size increased the number of intersections between strands, leading to more regions with elevated von Mises stress (Fig. 10). This resulted in a slight increase in OSS_avg_, as shown in Fig. 9a. However, the changes in WSS_avg_ and OSS_avg_ during step 2 were less pronounced than those observed in step 1, leading to smaller variations in cell differentiation as a function of pore size (Figs. 11 and 12). Thus, it can be concluded that porosity has a more significant impact on cell differentiation than *D*_pore_.

Previous studies on the optimal design of bone scaffolds have typically considered a pore size range of approximately 200–800 µm as a constraint in optimization models (Perez and Mestres 2016; Foroughi et al. 2025). Our findings in step 2 indicate that the investigated scaffolds fall within this range, with larger pore diameters demonstrating enhanced bone cell differentiation. As pore size increases, the number of adhering cells rises, leading to improved cell proliferation and viability (Sun et al. 2025). However, excessively large pores may reduce the effective surface area available for cell attachment, potentially compromising cell adhesion and diminishing the implant’s load-bearing capacity (Zhang et al. 2019; Lv et al. 2022; Sun et al. 2025).

The influence of OSS and WSS on the calculated maximum stimulus (${S}_{\text{Max}2}$) was analyzed separately for the scaffold with the largest pore size (S2-H8-V8) and the scaffold with the smallest pore size (S2-H11-V11). For S2-H8-V8, OSS contributed 42.2%, while WSS accounted for 57.8% of the total stimulus. In contrast, for S2-H11-V11, OSS contributed 38.2%, and WSS had a larger share of 61.8%. This indicates that as the pore diameter decreases while maintaining constant porosity, fluid shear stress plays a more dominant role in mechanical stimulation compared to structural strain.

### Comparison with experimental works

Experimental studies show that the scaffold structure significantly influences key characteristics in cartilage and bone tissue engineering, such as the promotion of cell differentiation or tissue formation as well as mechanical properties, biodegradability, and biocompatibility (Zhou et al. 2023).

Previous in vitro research indicated that chondrocytes exhibit optimal proliferation and extracellular matrix (ECM) production in scaffolds with pore sizes between 250 and 500 μm (Lien et al. 2009). This finding is consistent with our investigated scaffolds in step 2 (Table 3).

Moreover, an in vivo study by Zhang et al. (2022) investigated the effect of pore size on osteoinduction two months after implantation in the animal. Their experimental results showed that new bone tissue formation was greatest in scaffolds with 600 μm pores, followed by those with 800, 400, and 200 μm pores, respectively. Since larger pore sizes in their study corresponded to higher porosity, it can be inferred that greater porosity generally promotes increased bone formation. This observation aligns qualitatively with our simulation results for bone differentiation in step 1. Similarly, a recent study by Zhang et al. (2024) on titanium scaffolds with diamond lattice structures demonstrated that in vivo bone formation was higher in scaffolds with greater porosity and permeability.

### Limitations and future works

A limitation of this study arises from the use of a two-step approach instead of applying a full factorial design of experiments. If the modification strategy used in step 2 had been applied not just to S1-H9-V9 but to all scaffolds in step 1, the total number of scaffold architectures designed in step 2 could have been 15 instead of 3 (Fig. 8). However, this would have made our analysis much more computationally intensive and could have included scaffold designs that were not feasible with DIW due to strand diameters being either too large or too small, or pore sizes that exceeded the limited resolution of the DIW process.

Computer optimization algorithms have been employed to enhance scaffold designs for bone tissue regeneration (Boccaccio et al. 2016; Rodríguez-Montaño et al. 2020; Perier-Metz et al. 2022; Josephson and Morgan 2024). As an example, the study done by Perier-Metz et al. (2022) utilized the “particle swarm optimization” (PSO) algorithm to predict an optimal titanium scaffold design, achieving 96% regenerated bone within the scaffold pores at a porosity of 85%. However, applying the same algorithm to optimize soft scaffolds resulted in only 67% regenerated bone in the scaffold pores with a porosity of 24% (Perier-Metz et al. 2022). Implementing such optimization strategies based on our modeling approach could be beneficial in future studies. Nevertheless, considering the producibility limitations of hydrogel scaffolds using the DIW method, it will be challenging to implement optimization algorithms. Future studies could also develop Machine Learning (ML) models to find the correlation between scaffold architectures and mechanobiological parameters (Ibrahimi et al. 2024). However, training these ML models would require a sufficient number of scaffold samples (Ibrahimi et al. 2024). In addition to these numerical advancements, future investigations should validate our numerical predictions of cell differentiation through in vitro experiments using a compression bioreactor.

In the present model, material properties were assumed to remain constant; therefore, the predicted cell phenotypes do not change when additional loading cycles are simulated. In other words, the model accounts only for the scaffold’s homeostatic state, while transient processes influencing cell differentiation are not considered. Future studies should incorporate dynamic changes in material properties and scaffold morphology due to neo-tissue formation, updating these characteristics over multiple loading cycles during long-term stimulation. This approach would allow the model to more accurately reflect real in vitro and in vivo alterations in scaffold properties, leading to improved predictions based on the computed mechanical stimuli.

## Conclusion

In this study, we employed an FSI-based model to improve the geometry of a 3D structured scaffold for cartilage cell differentiation through a two-step modification process. In step 1, we adjusted the number of strands in each horizontal layer to determine the optimal horizontal span, identifying a design with 9 strands per layer that enhanced cartilage cell differentiation on the scaffold surface by approximately 15%. In step 2, we further refined the selected scaffold from step 1 by altering the pore dimensions while maintaining nearly constant porosity. This modification resulted in an additional increase in cartilage differentiation of about 2.3%. Future research can extend this modeling approach by incorporating optimization algorithms to identify the most effective architectural design for cartilage cell differentiation. However, challenges such as the manufacturability of hydrogel scaffolds using the DIW method and the computational costs associated with iterative transient FSI simulations to update geometrical parameters need to be addressed.

## Supplementary Information

Below is the link to the electronic supplementary material.

Supplementary file1 (PDF 813 kb)

## Data Availability

No datasets were generated or analyzed during the current study.
