# Supplementary material for: Numerical study of the structural design influence on cartilage cell differentiation in mechanically stimulated hydrogel scaffolds using an FSI-based model
Source: Biomech Model Mechanobiol. 2025 Jun 15;24(4):1417–33. doi: 10.1007/s10237-025-01976-1 (PMC12246015; doi:10.1007/s10237-025-01976-1)
Supplement: Supplementary file 1 — Supplementary file1 (PDF 813 kb) [file 10237_2025_1976_MOESM1_ESM.pdf]

Supplementary Material for:

Numerical Study of the Structural Design Influence on Cartilage Cell Differentiation in Mechanically Stimulated Hydrogel Scaffolds Using an FSI-Based Model

Pedram Azizi[1,2*], Christoph Drobek[1], Hermann Seitz[1]

[1] Chair of Microfluidics, Faculty of Mechanical Engineering and Marine Technology, University of Rostock, Rostock, Germany

[2] Chair of Electromagnetic Field Theory, Institute of General Electrical Engineering, Faculty of Computer Science and Electrical Engineering, University of Rostock, Rostock, Germany

*** Correspondence:**
Corresponding Author
Pedram.azizi@uni-rostock.de ;

1 Geometry Description

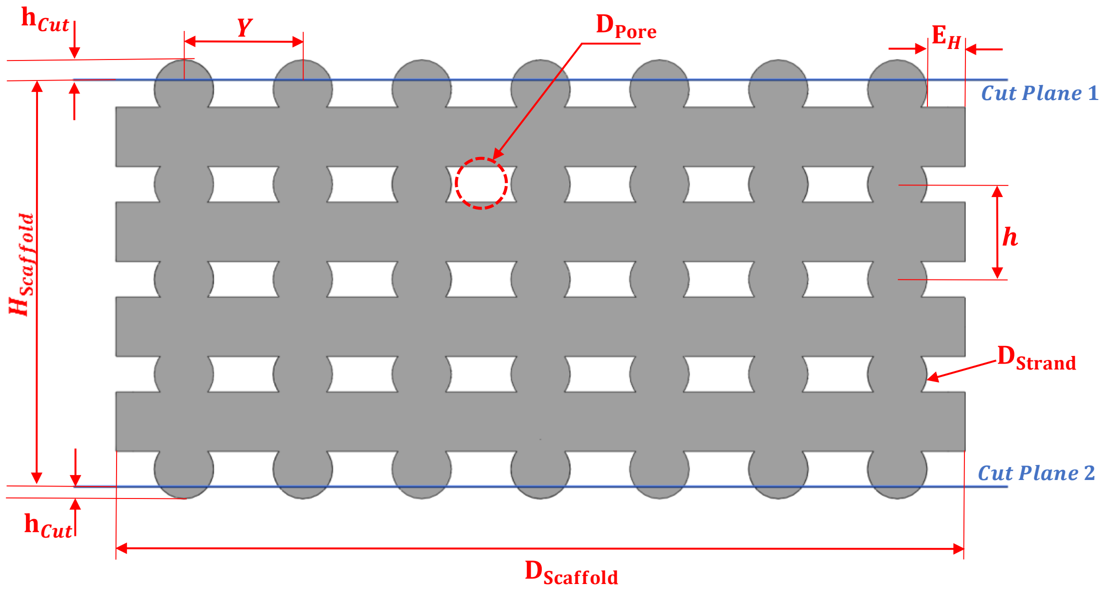

Fig. S1 A sectional view of the original scaffold, with the geometric parameters detailed in Table S1

Table S1 Definition of geometrical parameters and their corresponding values of the original scaffold
* The cutting planes were used to remove a small part of the first and last layers of the scaffold. This process ensured stable contact between the scaffold and the support as well as between the scaffold and the piston.
** The diameter of an imaginary circle that encloses the smallest pore formed by four strands within the scaffold.

Geometric Parameter	Definition	Value [mm]
$\boldsymbol{H_{Scaffold}}$	Scaffold height	4.8
$\boldsymbol{D_{Scaffold}}$	Scaffold diameter	10
$\boldsymbol{D_{Strand}}$	Strand diameter	0.7
$\boldsymbol{Y}$	Horizontal span	1.4
$\boldsymbol{h}$	Vertical span	1.12
$\boldsymbol{E_H}$	Horizontal edge of the scaffold	0.45
$\boldsymbol{h^{*}_{Cut}}$	Distance of the cutting planes to the piston/ Support	0.19
$\boldsymbol{D^{**}_{Pore}}$	minimal equivalent pore diameter	0.63

2 Step 1: Variation of Y

The horizontal span (Y) was defined as a function of the number of strands in each horizontal layer (N_H) using the following geometrical equation:

$$Y = \frac{D_{Scaffold} - D_{Strand} - 2E_H}{N_H - 1} \qquad \textbf{Eq.S1}$$

where $D_{scaffold}$, D_{strand}, and E_H are the geometrical constants (Table S1). This equation was used to change the geometry of the scaffold in step 1.

3 Step 2: Variation of h and D_{strand}

The pore diameter (D_{pore}) can be calculated using a geometrical equation as a function of $D_{scaffold}$, D_{strand}, and h:

$$D_{Pore} = \sqrt{\frac{4h_1\left(Y - \sqrt{{D_{Strand}}^2 - h_1^2}\right) - 2\left({D_{Strand}}^2 . sin^{-1}(\frac{h_1}{D_{Strand}}) - h_1 . \sqrt{{D_{Strand}}^2 - {h_1}^2}\right)}{\pi}} \qquad \textbf{Eq.S2}$$

Where h_1 is defined using the following equation:

$$h_1 = h - D_{Strand} \qquad \textbf{Eq.S3}$$

Geometric constraints that remained constant during the modifications in step 2:

$$x_1 = \frac{h_{cut}}{D_{Strand}} \qquad \textbf{Eq.S4}$$

$$x_2 = \frac{h}{D_{Strand}} \qquad \textbf{Eq.S5}$$

In step 2, these two ratios ($x_1 = 0.27$ and $x_2 = 1.6$) were maintained constant based on the original design S0-HL7-VL9. These assumptions were necessary to preserve the same $H_{scaffold}$ as the original design while aligning h proportionally with the D_{strand} and not arbitrarily chosen. Specifically, keeping x_1 constant ensures that the position of the cut plane (h_{cut}) remains proportional to D_{strand}, and with x_2 as a constant, changing h necessitates a corresponding adjustment in D_{strand}. Considering these constants and the vertical geometric relationships among the parameters defined in Fig. S1, h can be determined as a function of the number of scaffold layers (N_v) from:

$$h = \frac{2H_{Scaffold}}{N_v - (\frac{4x_1 - 2}{x_2}) - 1} \qquad \textbf{Eq.S6}$$

where $H_{scaffold}$, x_1, and x_2 are constant, and N_v is variable.

To apply the optimal value of Y from step 1 ($Y_{Optimum}$) in step 2, an additional constant was defined as:

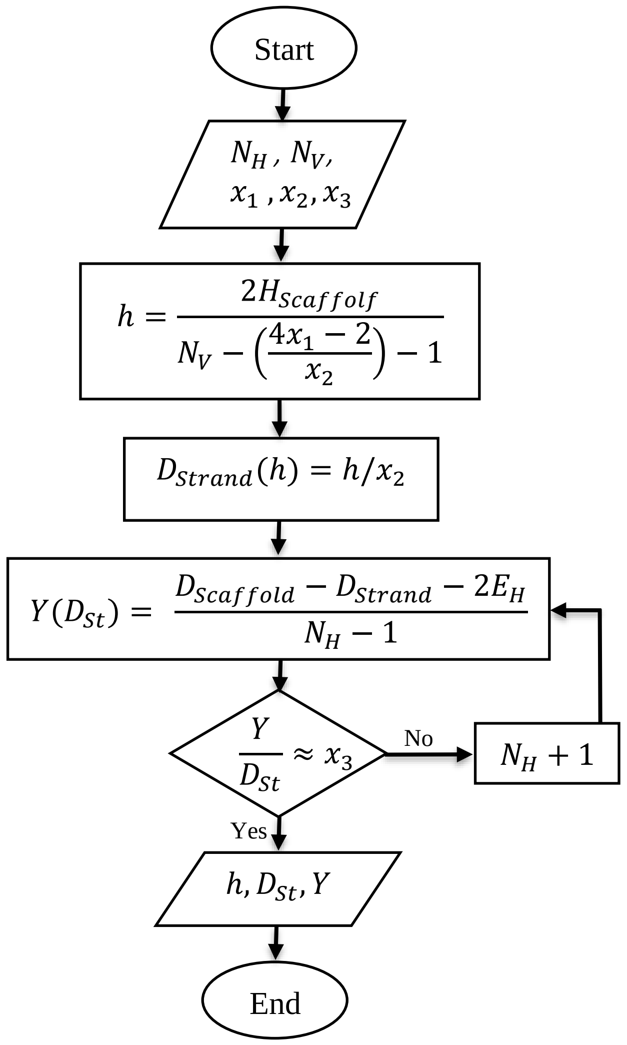

Fig. S2 Step 2 of scaffold optimization involves analyzing the impact of h by varying the number of layers in the vertical direction

$$x_3 = \frac{Y_{Optimum}}{D_{St}} \qquad \textbf{Eq.S7}$$

The specific value for this constant was determined based on the scaffold selected in step 1 (S1-H9-V9) and was equal to 1.5.

The strategy employed to optimize the scaffolds in step 2 is summarized in a flowchart depicted in Fig. S2.

4 Mesh Independence Study

A mesh independence study was conducted on the original design S0-HL7-VL9 to determine the appropriate number of elements. The examined mesh configurations are listed in Table S2, with Mesh 1 being the coarsest and Mesh 5 the finest.

Table S2 Mesh independence analysis using 5 different mesh configurations under 5% compression. Mesh 4 was chosen for all subsequent simulations in this study. The 'Mesh Details' column presents a detailed view of the conformal meshing, highlighting both the scaffold (in gray) and the pores (in brown)

Mesh Size	Number of FE Model Elements	Number of CFD Model Elements	Rate of Increase of CFD Elements	Total Simulation Time: CFD + FE Model + Coupling	Mesh Details
**Mesh1**	311,501	615,386	-	5 h + 59 min	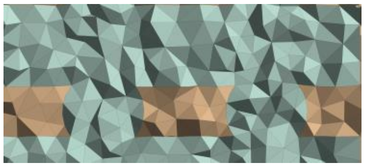
**Mesh2**	526,866	924,169	1.50	15 h + 52 min	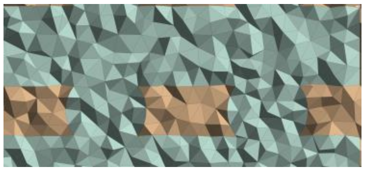
**Mesh3**	862,472	1,381,513	1.49	1d + 8 h + 3 min	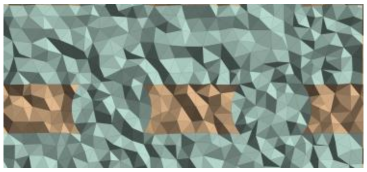
**Mesh4**	1,436,128	2,093,257	1.52	4 d + 10 h + 30 min	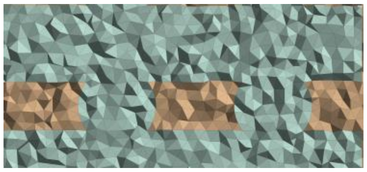
**Mesh5**	2,435,890	3,084,554	1.47	1 w + 5 d + 17 h + 9min	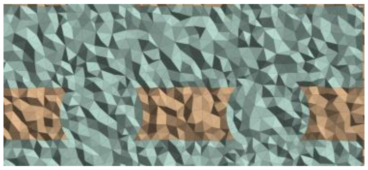

Fig. S3 displays the results of the mesh independence study. The relative error for WSS_{Avg} (Fig. S3C) and OSS_{Avg} (Fig. S3D) for Mesh 4, compared to Mesh 5, was below 5% most simulation times. Moreover, the absolute error in cell differentiation for Mesh 4 was approximately 1% relative to Mesh 5 (Fig. S3F). Therefore, Mesh 4 configuration was implemented for all designed geometries in both step 1 and step 2. The curvature normal angle of mesh 4 on the scaffold was set to 20°, while other domain regions used an angle of 60°. Furthermore, the element size on the scaffold was chosen as 0.1mm, whereas a value of 0.6mm was selected for other parts.

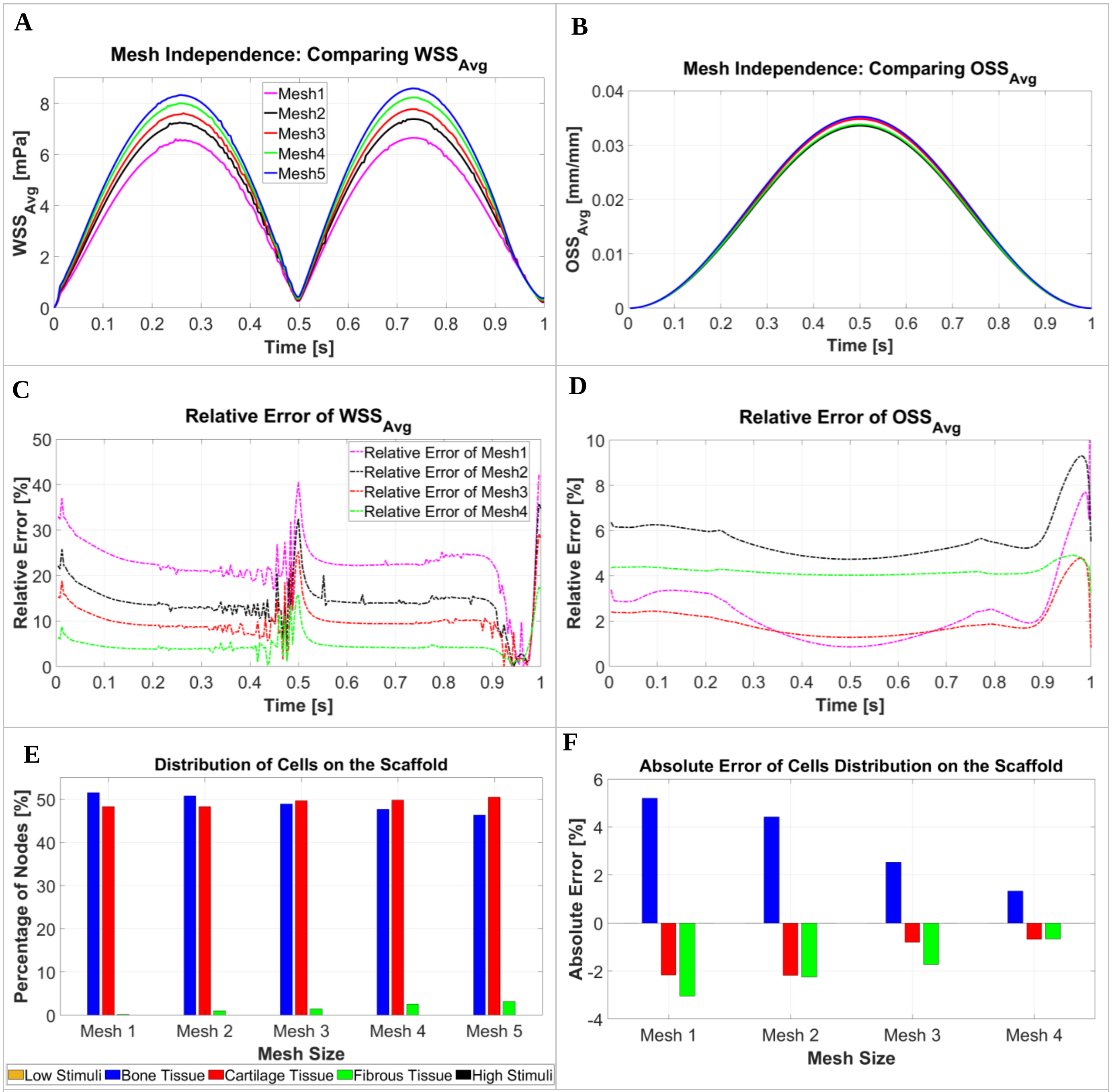

Fig. S3 Mesh independence study on WSS_{Avg} (**A**) and OSS_{Avg} (**B**). The number of elements increased gradually from Mesh 1 to Mesh 5, with all simulations performed under compression of 5%. The relative error of WSS_{Avg} compared to Mesh 5 (**C**) and the relative error of OSS_{Avg} compared to Mesh 5 (**D**). Cell differentiation for different mesh configurations (**E**) and the absolute error of cell differentiation relative to Mesh 5 (**F**). All the cell differentiation results are calculated at the time point when the model with the Mesh 4 configuration reached its maximum S_{Avg} value

5 Time-Step Size Sensitivity

Following the selection of Mesh 4, a time-step size sensitivity study was conducted to evaluate the effects of different time-step sizes, denoted as large (0.01s), medium (0.007s), and small (0.004s), on the simulation results (Fig. S4). The influence of studied time-step sizes on WSS_{Avg} and OSS_{Avg} was negligible (Fig. S4A-B). The absolute error in cell differentiation was approximately 0.2% for the largest time-step size (Fig. S4D). Therefore, the large time-step size was selected for all simulations in this study.

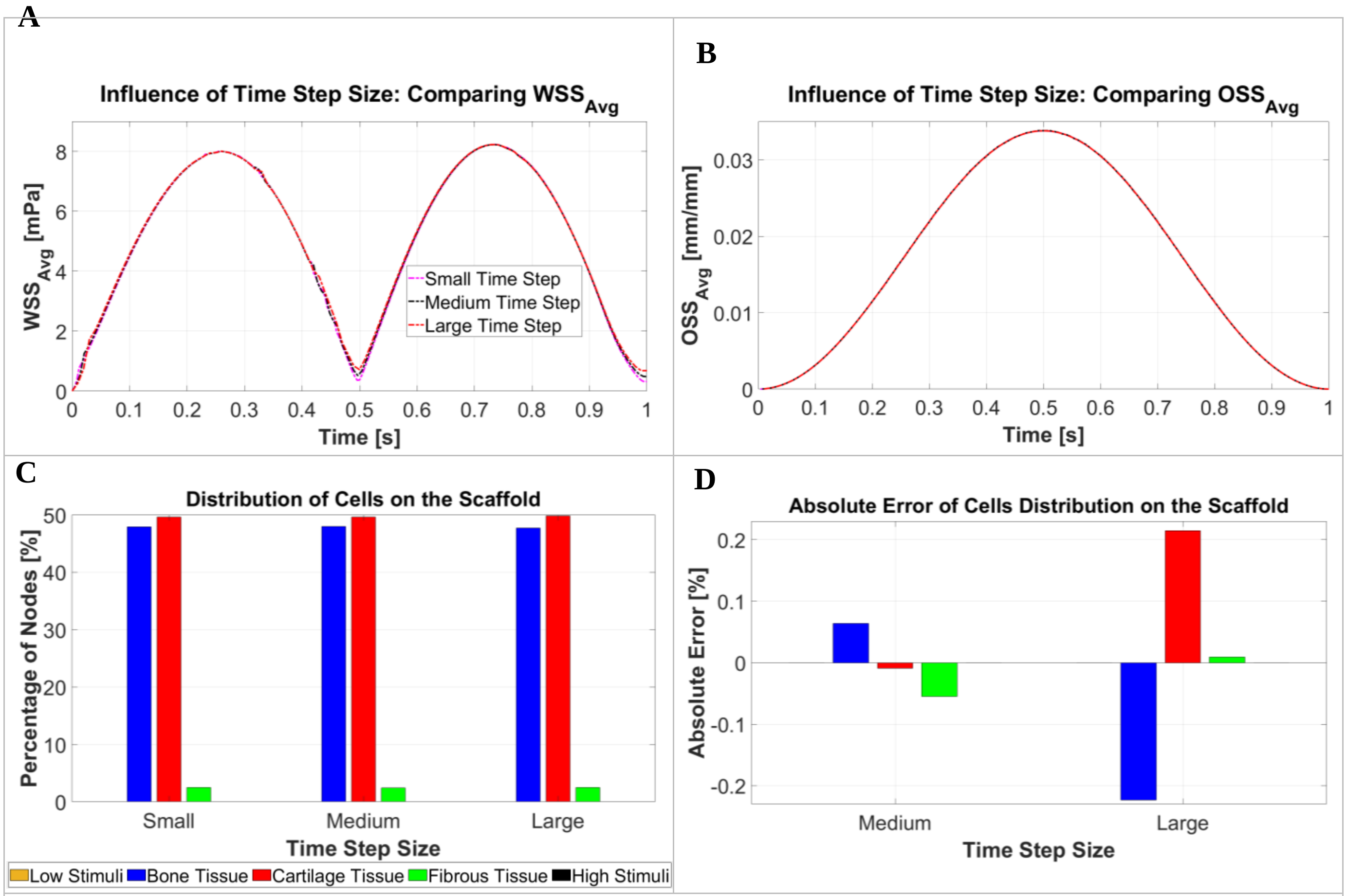

Fig. S4 Time-step size sensitivity study on WSS_{Avg} (**A**) and OSS_{Avg} (**B**). All simulations were conducted using Mesh 4 under 5% compression. Cell differentiation for three different time-step sizes (**C**) and the absolute error of cell differentiation relative to the smallest time-step size (**D**). All cell differentiation results were calculated at the time point when the model with the largest time-step size reached its maximum S_{Avg} value
